# Microstructure Evolution and Performance Improvement of Hypereutectic Al–Mg_2_Si Metallic Composite with Ca or Sb

**DOI:** 10.3390/ma13122714

**Published:** 2020-06-15

**Authors:** Min Zuo, Boda Ren, Zihan Xia, Wenwen Ma, Yidan Lv, Degang Zhao

**Affiliations:** School of Materials Science and Engineering, University of Jinan, Jinan 250022, China; rbd18863515609@163.com (B.R.); xzh778888@163.com (Z.X.); mwwdeyouxiang@126.com (W.M.); lyd1169704770@163.com (Y.L.); mse_zhaodg@ujn.edu.cn (D.Z.)

**Keywords:** Al–Mg_2_Si composite alloy, chemical modifier, microstructure, Brinell hardness, heterogeneous nucleation behavior

## Abstract

In this article, the modification effects on Al–Mg_2_Si before and after heat treatment were investigated with Ca, Sb, and (Ca + Sb). In comparison with single Ca or Sb, the samples with composition modifiers (Ca + Sb) had the optimal microstructure. The sample with a molar ratio for Ca-to-Sb of 1:1 obtained relatively higher properties, for which the Brinell hardness values before and after heat treatment were remarkably increased by 31.74% and 28.93% in comparison with bare alloy. According to differential scanning calorimetry analysis (DSC), it was found that the nucleation behavior of the primary Mg_2_Si phase could be significantly improved by using chemical modifiers. Some white particles were found to be embedded in the center of Mg_2_Si phases, which were deduced to be Ca_5_Sb_3_ through X-ray diffraction (XRD) and field-emission scanning electron microscope (FESEM) analyses. Furthermore, Ca_5_Sb_3_ articles possess a rather low mismatch degree with Mg_2_Si particles based on Phase Transformation Crystallography Lab software (PTCLab) calculation, meaning that the efficient nucleation capability of Ca_5_Sb_3_ for Mg_2_Si particles could be estimated.

## 1. Introduction

Recently, aluminum matrix composites (AMCs), especially Mg_2_Si-particle-reinforced aluminum matrix alloys, have attracted considerable attention due to their high specific strength, excellent wear, and anticorrosion resistance in structural components in the aerospace and automotive fields [1,2,3]. As an advanced intermetallic compound, Mg_2_Si possesses excellent properties, including high hardness (4.5 × 10^9^ N m^−2^), low density (1.99 × 10^3^ kg m^−3^), low thermal expansion coefficient (7.5 × 10^−6^ K^−1^), and a suitable elastic modulus (120 GPa), meaning it has been regarded as an intriguing candidate for use in structural applications [4,5]. As is known to all, the material performance is closely related to the configuration state [6,7]. Based on this, the question of how to prepare the controllable structural configuration of metallic alloys has been the focus of a number of studies. 

As for Al–Mg_2_Si alloys, the modification and refinement of Mg_2_Si phases have become especially important topics, in which coarse primary Mg_2_Si dendrites [8] and Chinese script eutectic Mg_2_Si [9,10] would seriously weaken the mechanical properties of systems. Over the past few years, many researchers have developed various routes to achieve the optimization effect of the Mg_2_Si phase, such as melt superheating [11], hot extrusion [12,13], ultrasonic melt treatment [14], rapid solidification [15], and heat treatment [16,17]. Besides these methods, chemical modifier treatments have been found to be much easier to operate with high efficiency, such as with Na [18], Sr [19], Be [20], Gd [21], La [22], and Y [23]. Farahany et al. [24] reported that a Bi additive also had a significant effect on the morphologies of Al_2_Cu (θ), Al_5_FeSi (β), and Al_5_Cu_2_Mg_8_Si_6_ (Q) phases, in addition to an excellent modification effect on primary and eutectic Mg_2_Si in Al–20Mg_2_Si–2Cu composites. Alizadeh and Mahmudi [25] found that Sb could change the morphologies of Mg_2_Si particles in Mg–4Zn–2Si alloys from Chinese script to more rounded edge types, resulting in better plasticity for shear deformation. 

Based on the references above, it was found that most of chemical modifiers only work on one of the two Mg_2_Si phases (primary Mg_2_Si or eutectic Mg_2_Si), with limited effect. Based on this, the complex modification treatment deserves more attention, owing to the considerable potential for the microstructural optimization of metallic alloys. Lv et al. revealed [26] that primary Mg_2_Si particles were significantly refined from 95 μm to 10 μm with 2.0% Sb and 0.2% Bi, for which the Brinell hardness was increased up to 134.67 after heat treatment. Based on the rather low planar mismatch δ between (111)_Mg__2Si_ and (0001)_Mg__3Sb__2_ of 1.79%, Mg_3_Sb_2_ could act as the heterogeneous nucleation site for primary Mg_2_Si, and with the cooperative absorption behavior of Bi on the growth front, the primary Mg_2_Si dendrites would be obviously refined. Zha et al. [27] reported that an enhanced synergistic modification effect of Sb and Zr could be obtained for Al–20Mg_2_Si alloys. Mg_3_(Sb, Si)_2_ particles could serve as the nuclei of primary Mg_2_Si and Zr, further improving the refinement effect through the decrease of the solidification temperature interval of the primary particles. Researchers still need to do a lot of work to identify the interaction effects between chemical modifiers, such as promotion, inhibition, or no influence.

Recently, the complex additives of Ca and Sb into aluminum alloys containing Mg_2_Si particles have drawn much attention. Yu et al. [28] revealed that CaSb_2_ particles could serve as heterogeneous nucleation sites for primary Mg_2_Si and Ca elements adsorbed on the growth front could restrict the growth rate of Mg_2_Si. Due to the large electronegativity difference between Ca and Sb of 1.05, various Ca–Sb compounds could form. In this paper, the composite modifications of Ca and Sb with different atomic ratios in Al–20Mg_2_Si alloys were applied in this process. The effects of single Ca and Sb modifiers on the microstructure and mechanical properties of composites before and after heat treatment were also reported. The novel complex modification of Ca and Sb would open significant new opportunities for microstructural control and property enhancement of aluminum alloys containing Mg_2_Si reinforcements. 

## 2. Experimental Procedures

Commercially pure Al ingot (99.97%), pure Mg ingot (99.89%), and Al–24Si master alloy were used as raw materials to prepare basic Al–12.7Mg–7.4Si alloys, which correspond to Al–20Mg_2_Si alloys (all compositions are in wt %, unless otherwise stated). Ca in the form of Al–29Ca master alloy and pure Sb ingot (98.25%) was added into Al–20Mg_2_Si to investigate the interaction effects of modifiers.

Melt modifications of Al–20Mg_2_Si alloys were carried out as follows. By using an electrical resistance furnace (SG2–7.5–12, Longkou Electric Furnace Factory, Longkou, China ), about 300 g of basic alloy was remelted in a graphite crucible (φ94 × 154 mm) (Jinda Crucible Factory, Tianjin, China )at 780 °C for 30 min. Subsequently, single modifier of Ca or Sb and a composite modifier (Ca + Sb) were added into the melt and mechanically stirred with a graphite rod for 10 min in order to obtain uniform dispersion. During the modification process, the melt temperature was monitored by a thermocouple and assisted by a K-model handset thermocouple (Wuxi Ruiwen Automation Instrument Co. LTD, Wuxi, China). Finally, the melt was poured into a permanent mold (70 × 35 × 20 mm) preheated to 200 °C. For the first group of modification treatments, the addition level of Ca was 0.15%. The second group of experiments was used to treat base alloys with various amounts of Sb (i.e., 0.23%, 0.46%, and 0.91%). Thirdly, the interaction influence of the composite modifier (Ca + Sb) on the microstructure of the base alloy was studied. In order to obtain more information, three molar ratios of Ca and Sb were evaluated, including 2:1 (0.15% Ca, 0.23% Sb), 1:1 (0.15% Ca, 0.46% Sb), and 1:2 (0.15% Ca, 0.91% Sb).

The Al–20Mg_2_Si samples were heat-treated following the T6 procedure, which involves treating the solution at 510 °C for 4 h followed by water quenching and then artificial aging at 175 °C for 8 h. Metallographic specimens were cut from the midsection of each sample and then mechanically ground and polished through standard routines. In order to evaluate the microstructures of alloys, the average sizes of primary Mg_2_Si particles were obtained from at least ten areas of specimen’s metallograph, and from each area about ten primary Mg_2_Si particles were chosen. The microstructural characterization of Al–20Mg_2_Si samples were determined with an optical microscope (4XC, Shanghai Caikon Optical Instrument Co. LTD, Shanghai, China) and a field-emission scanning electron microscope (FESEM) (QUANTA FEG250, FEI, Hillsboro, OR, USA) assisted by an energy-dispersive X-ray spectrometer (EDS) (INCA Energy X-MAX-50X, OIMS, Oxford, UK). The phase compositions of basic alloys were determined using X-ray diffraction (XRD) (D8 ADVANCE, Bruker, Germany) with Cu-Kα in steps of 10°–80°. Meanwhile, the scanning rate and acquisition step were 4°/min and 0.02° (2θ), respectively. Brinell hardness values (HB) of Al–20Mg_2_Si composite alloys with different chemical modifiers were evaluated by a Brinell hardness tester with an applied load of 612.9 N pressed by a φ5 indenter (HBRVU-187.5, Shanghai Materials Testing Instrument Co. LTD, Shanghai, China). For each sample, the hardness values were obtained according to the average value from seven points. The melting and solidification behaviors of alloys were evaluated using differential scanning calorimetry (DSC) (DSC-404, Netzsch Inc., Selb, Germany) under argon flow protection within the range of 200–750 °C at a rate of 10 °C/s. The possible orientation relationships between intermetallic compounds in this system were developed with the Phase Transformation Crystallography Lab software (PTCLab Version 1.19.0) [29].

## 3. Results and Discussion

**Figure 1:**
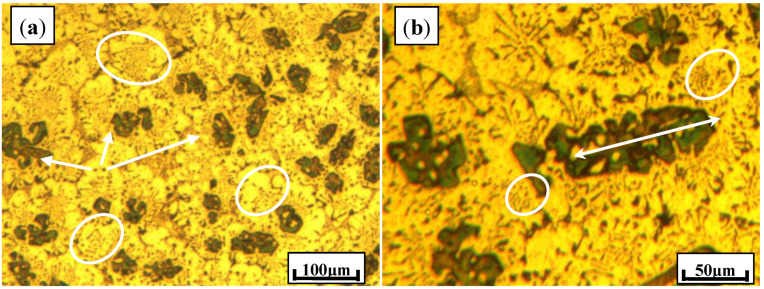
(**a**) typical microstructure of Al–20Mg_2_Si composites modified with 0.15% Ca; (**b**) partial enlarged drawing of this sample.

Figure 1 shows the typical microstructures of Al–20Mg_2_Si composite alloys modified with 0.15% Ca. As a typical surface active element, Ca restricts the growth rate of the Mg_2_Si phase, resulting in significant modification of particles. As the arrows indicate in Figure 1, primary Mg_2_Si particles were refined from more than 100.0 μm to about 44.1 μm, with standard deviation of 4.7 μm. However, some coarse Mg_2_Si dendrites were still detected with sizes up to 119.0 μm, as illustrated in Figure 1b. Furthermore, eutectic cell structures were obviously modified, as indicated by ellipses, with eutectic Mg_2_Si particles changing to short rod-like structures and dots. 

**Figure 2:**
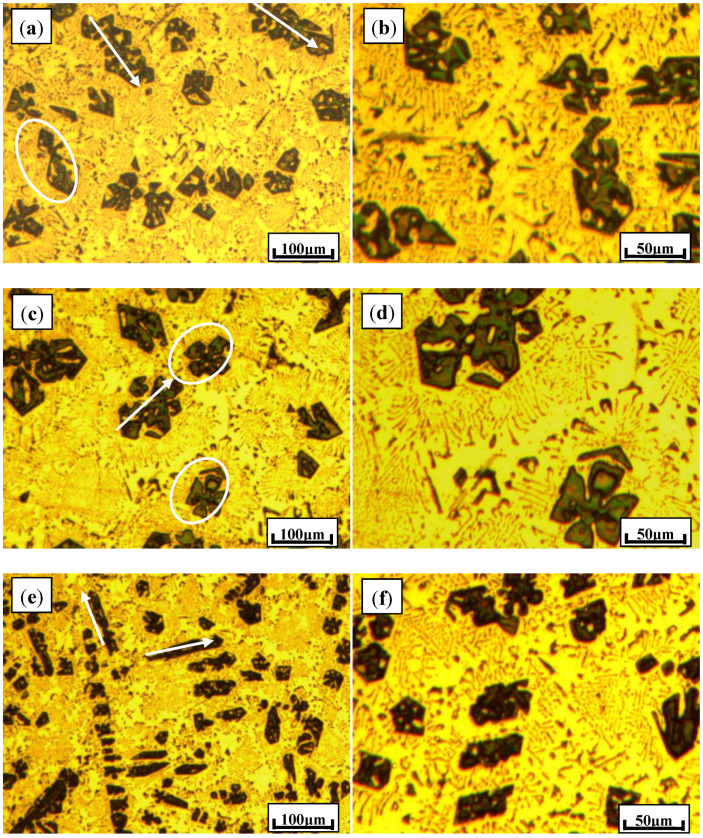
Typical microstructures of Al–20Mg_2_Si alloys with different addition levels of Sb: (**a**,**b**) 0.23%; (**c**,**d**) 0.46%; (**e**,**f**) 0.91%.

The typical microstructures of Al–20Mg_2_Si alloys modified with different addition levels of Sb are illustrated in Figure 2. It was found that Sb has an obvious influence on both phases of primary and eutectic structures. With the concentration of Sb increasing from 0.23% to 0.91%, the average sizes of primary Mg_2_Si particles were 54.2 μm (standard deviation of 4.8 μm), 67.1 μm (standard deviation of 4.0 μm), and 46.7 μm (standard deviation of 4.2 μm), respectively. As indicated by circles in Figure 2a, Mg_2_Si particles were obtained as corner-sharing octahedrons in three-dimensional spaces. Meanwhile, typical particles with four-petalled structures highlighted by circles were found, which were obtained from truncated octahedron Mg_2_Si along (001) planes, as shown in Figure 2c. Due to the enrichment of Al atoms at the center of the {111} planes, the hopper structure of the Mg_2_Si octahedron was formed. With the addition of Sb increasing to 0.91%, the microstructure of the Al–20Mg_2_Si composites was further optimized, with the eutectic cell structure being excellently modified.

**Figure 3:**
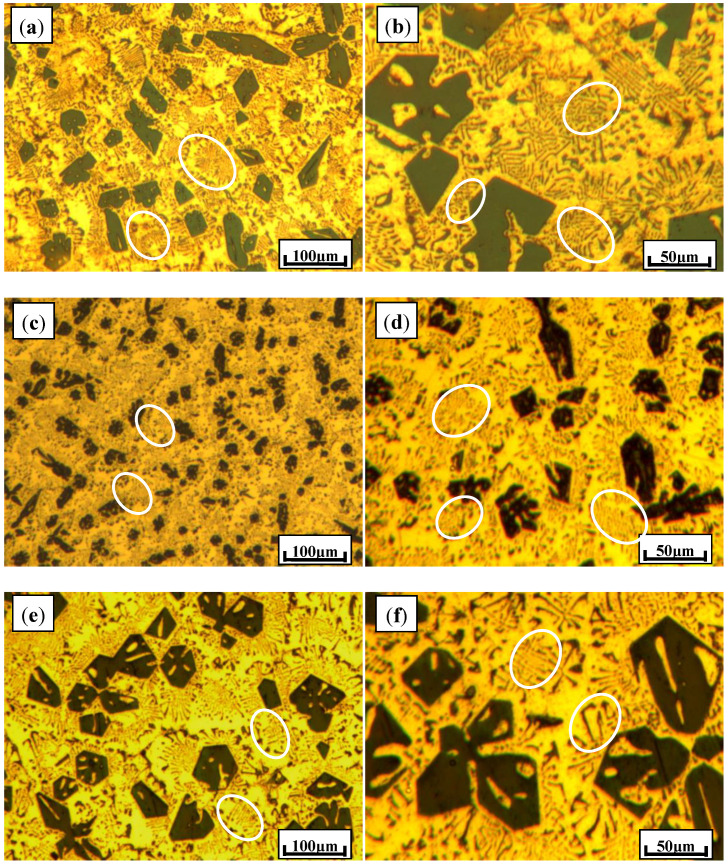
Typical microstructures of Al–20Mg_2_Si alloys modified by the complex modifier (Ca + Sb) with different molar ratios: (**a,b**) 2:1; (**c,d**) 1:1; (**e,f**) 1:2.

In comparison with modification efficiencies of single Ca and Sb, composite modifiers (Ca + Sb) with different molar ratios were added into Al–20Mg_2_Si alloys, such as 2:1, 1:1, and 1:2 ratios. As indicated in Figure 3, it was found that with a molar ratio of Ca-to-Sb of 1:1, the sample possessed better modification and refinement efficiency than the other two samples. In addition to the rather fine spot-like eutectic Mg_2_Si, primary Mg_2_Si particles were refined to 27.7 μm with standard deviation of 2.9 μm. With a higher concentration of Sb, the eutectic structure tended to be coarser, as illustrated in Figure 3e,f. The mean size of primary Mg_2_Si particles shown in Figure 3e was 55.8 μm. Furthermore, coarse flake-like eutectic Mg_2_Si compounds were also observed in this sample.

**Figure 4:**
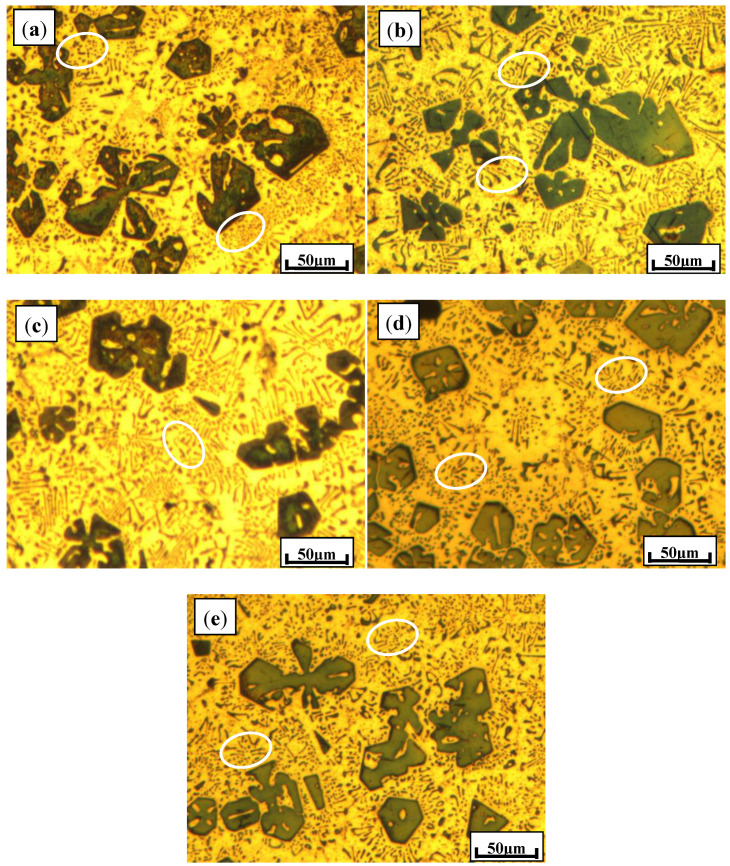
Typical microstructures of Al–20Mg_2_Si alloys with Ca or Sb modifiers after heat treatment: (**a**) 0.15% Ca; (**b**) 0.46% Sb; (**c**) 0.15% Ca; 0.23% Sb (molar ratio of 2:1); (**d**) 0.15% Ca and 0.46% Sb (molar ratio of 1:1); (**e**) 0.15% Ca and 0.91% Sb (molar ratio of 1:2).

In order to get more information, the typical microstructures of these composite alloys after T6 heat treatment were studied and are shown in Figure 4. In comparison with single Sb addition, eutectic Mg_2_Si compounds with a Ca modifier after heat treatment were finer. By modifying the compositions of Ca and Sb, the microstructures of the Al–Mg_2_Si alloys after heat treatment were further optimized. As clearly illustrated in Figure 4c–e, the sharp edges of primary Mg_2_Si polyhedrons became more rounded, with eutectic Mg_2_Si phases transforming from flakes into fine spot-like shapes simultaneously. The sample modified with 0.15% Ca and 0.46% Sb (molar ratios for Ca-to-Sb of 1:1) possessed the optimal microstructure, meaning that mechanical properties of metallic alloys could be better predicted.

**Figure 5:**
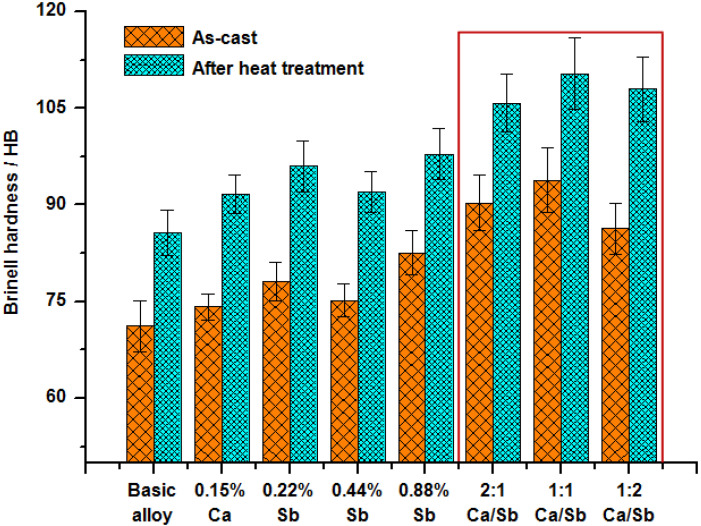
Brinell hardness values of Al–20Mg_2_Si alloys treated with different modifiers.

Figure 5 shows the Brinell hardness values of Al–20Mg_2_Si alloys treated with different modifiers. It can be seen that with single addition of Ca and Sb, the Brinell hardness values of as-cast compositions were increased by 4.21%, 9.69%, 5.62%, and 16.0% respectively, compared with the basic alloy. After heat treatment, the Brinell hardness values of alloys were all improved to a certain extent. The samples with complex modifications obtained relatively higher hardness values, especially the one with a Ca/Sb molar ratio of 1:1. The Brinell hardness values of this sample before and after heat treatment were about 93.8 and 110.4, increasing by 31.74% and 28.93%, respectively, in comparison with the bare alloy.

**Figure 6:**
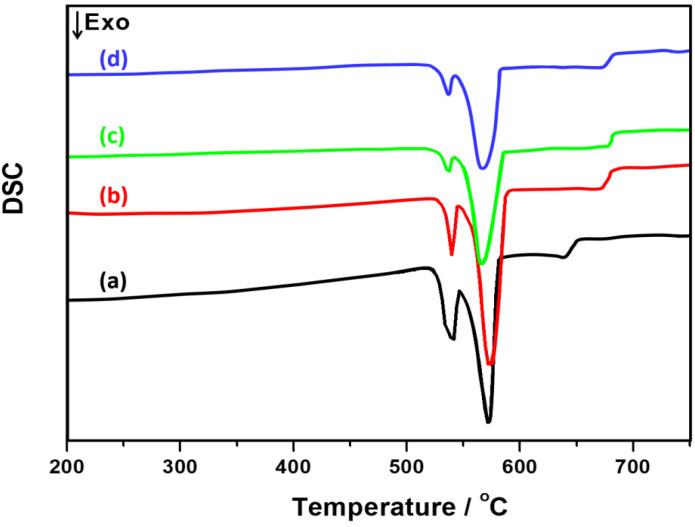
Differential scanning calorimetry (DSC) curves of Al–20Mg_2_Si alloys with different modifiers: (**a**) bare alloy; (**b**) 0.15% Ca; (**c**) 0.91% Sb; (**d**) 0.15% Ca and 0.23% Sb.

As we all know, the improvement of mechanical properties of metallic alloys is related to the optimization of the microstructure. With the addition of a chemical modifier, the solidification behavior of the Al–20Mg_2_Si alloys might be influenced, resulting in the evolution of their microstructures. Figure 6 shows the differential scanning calorimetry (DSC) curves of Al–20Mg_2_Si alloys with different chemical modifiers. As clearly indicated in Figure 6, the precipitation temperatures of primary Mg_2_Si phases in alloys modified by Ca or Sb were in the range of 675–683 °C with a deviation of 2 °C, which were relatively higher values than that of the bare alloy. The increase in precipitation temperature of the primary phase implies the decrease of the required undercooling temperature, indicating that the nucleation behaviors of primary phases were significantly improved. Furthermore, the initial precipitation temperatures of eutectic Mg_2_Si phases in these samples were 546, 545, 543, and 542 °C, respectively.

**Figure 7:**
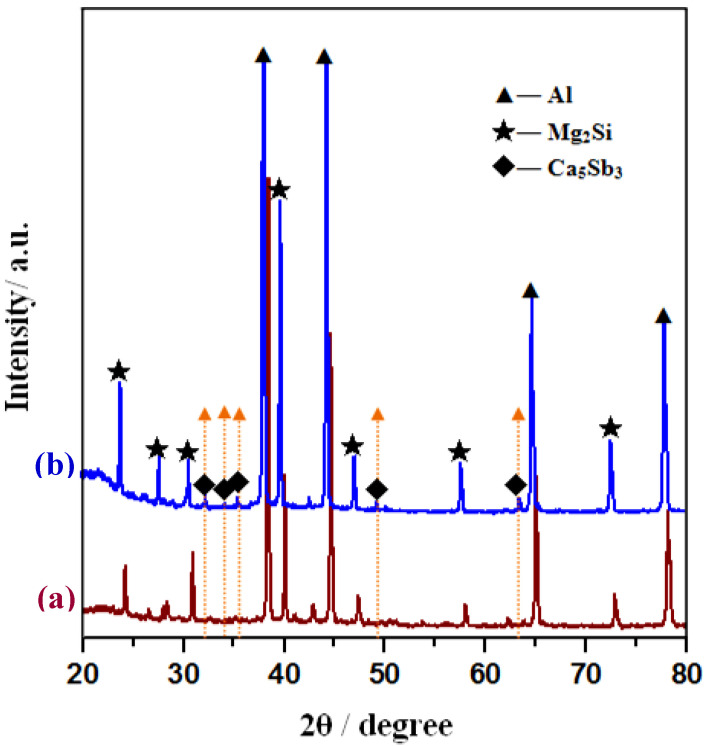
XRD patterns of as-cast Al–20Mg_2_Si alloys: (**a**) bare sample; (**b**) modified sample.

The corresponding XRD patterns of Al–20Mg_2_Si alloys before and after complex modifications of Ca and Sb are illustrated in Figure 7. In order to obtain clear diffraction peaks for possible compounds containing Ca or Sb, the concentrations of Ca and Sb were increased to about 1% and 3%, respectively, meaning that the molar ratio of Ca-to-Sb still remained at 1:1. Based on the XRD patterns, it could be found that besides Al and Mg_2_Si phases, some weak diffraction peaks were detected, which were determined to be Ca_5_Sb_3_ compounds based on ICSD #002065. As the orange arrows indicate in Figure 7, the diffraction lines exhibited peaks at 32.46°, 34.42°, 35.68°, 49.47°, and 63.62°, corresponding to (222), (420), (203), (304), and (642) reflections of Ca_5_Sb_3_ (orthorhombic, Pnma, a = 1.250 nm, b = 0.951 nm, c = 0.829 nm) respectively. The formation of Ca_5_Sb_3_ compounds might be the reason for the excellent complex modification efficiency of Ca and Sb in Al–20Mg_2_Si systems.

**Figure 8:**
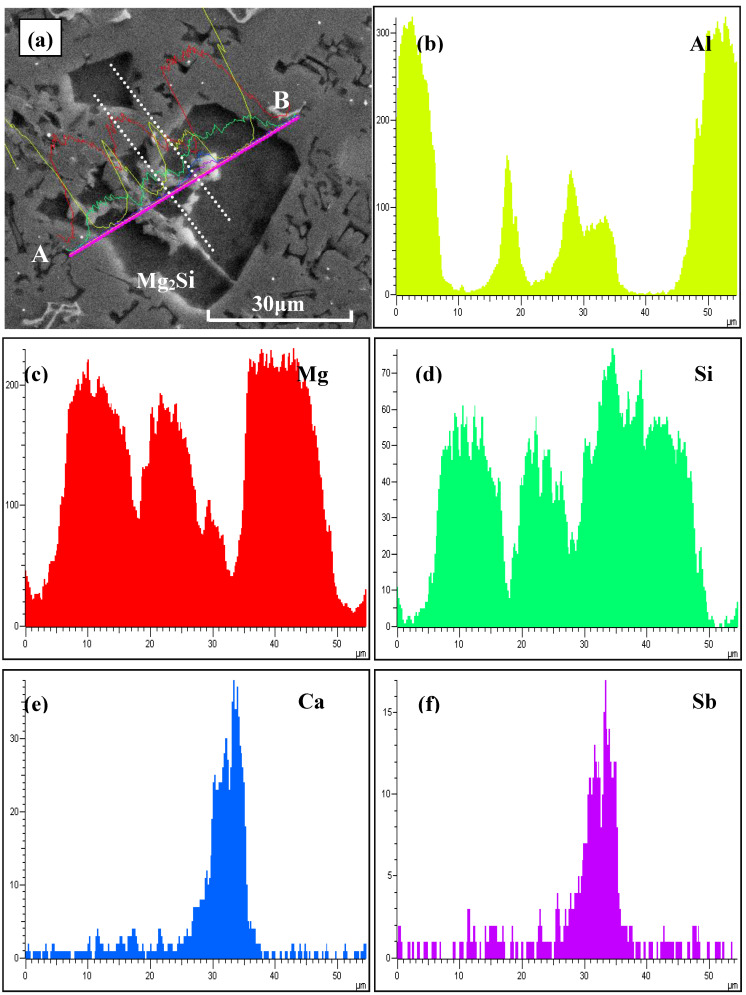
FESEM analysis of primary Mg_2_Si in Al–20Mg_2_Si alloys after modification by 0.15% Ca and 0.46% Sb: (**a**) microstructure; (**b**–**f**) the X-ray images for respective elements from A to B, including Al, Mg, Si, Ca, and Sb.

**Figure 9:**
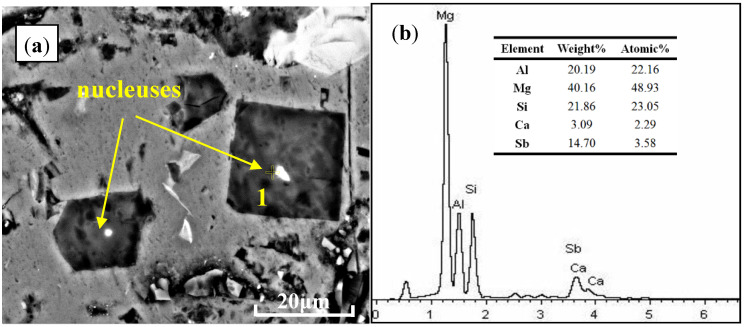
Typical FESEM analysis of Al–20Mg_2_Si metallic alloy with Ca and Sb chemical modifiers: (**a**) microstructure; (**b**) the corresponding EDS analysis of the nucleus 1# in (**a**).

The typical FESEM micrograph and corresponding EDS line scanning analyses along A–B are shown in Figure 8. It can be seen that there were some light grey particles embedded in the center of the primary Mg_2_Si particle. According to the X-ray images for respective elements, enrichment of Ca and Sb elements in the area between the two dotted lines was observed, which was confirmed to be the heterogeneous nucleation site for the primary Mg_2_Si phase during the solidification process. To obtain more details about this compound, a chemical composition analysis with an EDS test was carried out and is illustrated in Figure 9. From the FESEM image in Figure 9, the existence of light grey particles in the middle of primary Mg_2_Si particles was proven. Based on the EDS results, the atomic ratio of Ca and Sb elements with light grey particle was about 1.95, which was relatively closed to 1.67 in the Ca_5_Sb_3_ phase. In accordance with the XRD analysis, this enrichment of Ca and Sb in the center of Mg_2_Si provided further evidence of the heterogeneous nucleation effect of Ca_5_Sb_3_.

Yuan et al. [30] studied the microstructure refinement of Mg–Al–Zn–Si alloys with Sb and found that the morphological change of Mg_2_Si was attributed to Mg_3_Sb_2_ as the nucleation nuclei. As a surface-active element, Ca elements could be adsorbed on the {111} and {100} planes of Mg_2_Si particles, restricting the corresponding growth rates of these planes. Based on this, various stages of Mg_2_Si-truncated octahedrons enclosed by {111} and {100} lattice planes would be formed. The electronegativity is a typical chemical characteristic, which indicates the ability of an atom to attract other electrons to itself. The larger the difference between the electronegativity parameters of two atoms, the higher the tendency for them to form compounds. In this Al–Mg_2_Si system, Ca and Sb are likely to react with each other due to the large electronegativity difference of 1.05, resulting in the formation of various stoichiometric compounds [31]. Yu [32,33] investigated the crystallization behavior of primary Mg_2_Si in composites with different Ca/Sb molar ratios. In their study, the total mass concentration of Ca and Sb was 0.5%, in which the addition levels of Ca and Sb were determined to be within the range of 0.05%–0.25% and 0.25%–0.45%, respectively. Furthermore, it was found that CaSb_2_ and CaSi_2_ could affect the nucleation process of primary Mg_2_Si, resulting in the refinement of primary particles.

By using differential thermal analysis (DTA) and microstructure analysis, Niyazova et al. [34] first investigated the binary phase diagram of Ca–Sb and reported that three stable compounds could be formed, including Ca_3_Sb_2_, CaSb, and CaSb_3_. Okamoto [35] further researched the Ca–Sb phase diagram based on new DTA data and negative formation enthalpies of Ca_11_Sb_10_ and Ca_5_Sb_3_. In addition, Martinez-Rippoll and Brauer [36] deduced the Ca_3_Sb_2_ phase to be the stoichiometry of Ca_5_Sb_3_, and Leon-Escamilla and Corbett [37] refined the crystal structure of Ca_5_Sb_3_. Qin et al. [38] studied the thermodynamic modeling of the Ca–Sb system by using first-principles calculations. According to their research, the thermodynamic behaviors of different compounds in the Al–Mg–Si–Ca–Sb system could be discussed as follows. According to XRD and EDS analyses, the nucleation nuclei embedded in primary Mg_2_Si particles were deduced to be Ca_5_Sb_3_, which could be formed through the following Equation (1):

(1) $$5\mathrm{Ca}+3\mathrm{Sb}\to {\mathrm{Ca}}_{5}{\mathrm{Sb}}_{3}$$

The standard Gibbs free energy change ΔG could be obtained through the corresponding Equation (2):

(2) ΔG=−618918+207.28T

in which T is the temperature of the liquid melt in degrees Celsius. When the system temperature changed from 700 to 1000 °C, the ΔG values fell in the range of −411 to −473 kJ/mol, which proved the feasibility of this reaction due to the large negative values. In comparison with the formation reaction of CaSb_2_ [32], the precipitation of Ca_5_Sb_3_ possesses a much lower ΔG value, meaning a greater tendency for the formation of Ca_5_Sb_3_.

**Figure 10:**
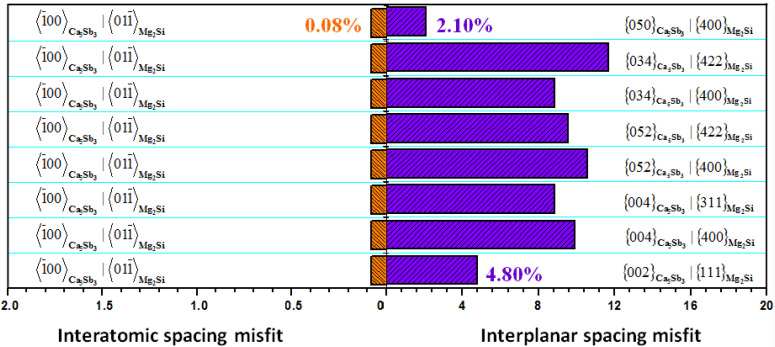
The corresponding interatomic spacing and interplanar spacing misfits between Ca_5_Sb_3_ and Mg_2_Si phases.

According to the edge-to-edge matching model [39,40], the Ca_5_Sb_3_ phase could act as a potential heterogeneous nucleation site for Mg_2_Si particles due to the low mismatch degree, which was obtained using Phase Transformation Crystallography Lab software (PTCLab). The detailed orientation relationships between Ca_5_Sb_3_ and Mg_2_Si phases are listed in Figure 10. Based on the calculations, the interatomic spacing misfit between ${\langle \bar{1}00\rangle }_{{\mathrm{Ca}}_{5}{\mathrm{Sb}}_{3}}$ and ${\langle 01\bar{1}\rangle }_{{\mathrm{Mg}}_{2}\mathrm{Si}}$ was rather low at 0.08%. Meanwhile, the corresponding interplanar spacing mismatches between ${\left\{050\right\}}_{{\mathrm{Ca}}_{5}{\mathrm{Sb}}_{3}}$/${\left\{400\right\}}_{{\mathrm{Mg}}_{2}\mathrm{Si}}$ and ${\left\{002\right\}}_{{\mathrm{Ca}}_{5}{\mathrm{Sb}}_{3}}$/${\left\{111\right\}}_{{\mathrm{Mg}}_{2}\mathrm{Si}}$ were merely 2.10% and 4.80%, respectively. From this, the efficient nuclear capability of Ca_5_Sb_3_ for Mg_2_Si particles could be estimated, which was further confirmed by the FESEM results shown in Figure 8 and Figure 9.

With the co-modification of Ca and Sb, the mechanical properties of Al–Mg_2_Si composite alloys were improved, which were attributed to the optimization of the microstructure. As for polycrystalline alloys, the effects of grain size on mechanical properties could be described by the Hall–Patch Equation (3). Based on the Hall–Patch model:

(3) $$H=H_{0}+{\mathrm{kD}}^{-1/2}$$

where H_0_ is the hardness of the bulk material, k is the constant in the relationship with the material characteristic, and D is the average grain size [41,42]. With the heterogeneous nucleation sites provided by Ca_5_Sb_3_, coarse primary Mg_2_Si dendrites were changed into fine particles. Additionally, the eutectic structure was also efficiently modified due to the existence of Ca. Through the efficient refinement of primary Mg_2_Si and modification of the eutectic structure, the hindrance ability of the grain boundaries as the obstacles for crack propagation would be significantly improved, resulting in the promotion of mechanical properties. 

## 4. Conclusions

In summary, a novel precipitation behavior control method with (Ca + Sb) was applied for microstructural control and performance improvement of Al–Mg_2_Si alloys. The modification mechanism of the complex modifier (Ca + Sb) in this alloy was also investigated.

- In comparison with single Ca or Sb modifiers, the optimum modification effect for the Al–Mg_2_Si alloy was obtained through the composite addition of (Ca + Sb), especially for the alloy with 0.15% Ca and 0.46% Sb (molar ratio of 1:1), which possessed an optimal microstructure configuration and mechanical properties;
- Through FESEM and XRD analyses, Ca_5_Sb_3_ compounds were observed in the center of the primary Mg_2_Si particles. Furthermore, the formation feasibility of Ca_5_Sb_3_ was also proven by thermodynamic calculation;
- According to DSC analysis, the precipitation of primary Mg_2_Si phases in Ca- or Sb-modified alloys was in the range of 675–683 °C, meaning that the precipitation behavior could be improved;
- Ca_5_Sb_3_ has a rather low mismatch degree with Mg_2_Si particles according to calculations using Phase Transformation Crystallography Lab software (PTCLab). The interatomic spacing misfit between ${\langle \bar{1}00\rangle }_{{\mathrm{Ca}}_{5}{\mathrm{Sb}}_{3}}$ and ${\langle 01\bar{1}\rangle }_{{\mathrm{Mg}}_{2}\mathrm{Si}}$ was rather low at 0.08%. Meanwhile, the corresponding interplanar spacing mismatches between ${\left\{050\right\}}_{{\mathrm{Ca}}_{5}{\mathrm{Sb}}_{3}}$/${\left\{400\right\}}_{{\mathrm{Mg}}_{2}\mathrm{Si}}$ and ${\left\{002\right\}}_{{\mathrm{Ca}}_{5}{\mathrm{Sb}}_{3}}$/${\left\{111\right\}}_{{\mathrm{Mg}}_{2}\mathrm{Si}}$ were merely 2.10% and 4.80%, respectively;
- Based on these findings, the efficient nucleation behavior of Ca_5_Sb_3_ for Mg_2_Si particles could be estimated, resulting in the improvement of the microstructure and mechanical properties of composite alloys.
